# Vertebral artery fusiform aneurysm geometry in predicting rupture risk

**DOI:** 10.1098/rsos.180780

**Published:** 2018-10-31

**Authors:** Xiukun Zhao, Nathan Gold, Yibin Fang, Shixin Xu, Yongxin Zhang, Jianmin Liu, Arvind Gupta, Huaxiong Huang

**Affiliations:** 1 Centre for Quantitative Analysis and Modelling (CQAM), The Fields Institute, Toronto, Ontario M5T 3J1, Canada; 2 The Fields Institute for Research in Mathematical Sciences, Toronto, Ontario M5T 3J1, Canada; 3 Department of Mathematics and Statistics, York University, Toronto, Ontario M3J 1P3, Canada; 4 Department of Neurosurgery, Changhai Hospital, Second Military Medical University, Shanghai, China; 5 Department of Computer Science, University of Toronto, Toronto, Ontario M5T 3J1, Canada

**Keywords:** aneurysm geometry, rupture risk prediction, machine learning

## Abstract

Cerebral aneurysms affect a significant portion of the adult population worldwide. Despite significant progress, the development of robust techniques to evaluate the risk of aneurysm rupture remains a critical challenge. We hypothesize that vertebral artery fusiform aneurysm (VAFA) morphology may be predictive of rupture risk and can serve as a deciding factor in clinical management. To investigate the VAFA morphology, we use a combination of image analysis and machine learning techniques to study a geometric feature set computed from a depository of 37 (12 ruptured and 25 un-ruptured) aneurysm images. Of the 571 unique features we compute, we distinguish five features for use by our machine learning classification algorithm by an analysis of statistical significance. These machine learning methods achieve state-of-the-art classification performance (81.43 ± 13.08%) for the VAFA morphology, and identify five features (cross-sectional area change of aneurysm, maximum diameter of nearby distal vessel, solidity of aneurysm, maximum curvature of nearby distal vessel, and ratio of curvature between aneurysm and its nearby proximal vessel) as effective predictors of VAFA rupture risk. These results suggest that the geometric features of VAFA morphology may serve as useful non-invasive indicators for the prediction of aneurysm rupture risk in surgical settings.

## 1. Introduction

Aneurysms, pathological dilation of blood vessel and weakening of the vessel wall, affect a significant portion of the adult population [1–3]. There exists an extensive literature with a large focus on possible correlations between the occurrence of aneurysms and hydrodynamic factors [4–6]. The findings are, however, inconsistent and sometimes conflicting. For example, high wall stress, low wall stress, low pressure, turbulence and flow instability have been identified as possible causes [7,8]. This is probably due to the fact that aneurysms are caused by multiple factors, with the exact cause not fully understood. Left untreated, some aneurysms may grow and rupture, causing uncontrollable haemorrhage. On the other hand, treatment also carries a certain risk, sometimes also causing undesirable consequences. Therefore, it is a life-and-death decision whether and what surgical intervention should be performed. To help surgical decision-making, it is desirable if certain features of aneurysms correlated to the likelihood of rupture can be identified.

There are two main types of aneurysms, saccular and fusiform, characterized by distinct morphologies. In saccular aneurysms, the contour/circumference of aorta remains intact, and is mostly uninvolved, with an eccentricity that involves only a part of the vessel wall contour. Fusiform aneurysms, conversely, result in complete distortion of the aortic contour, with a concentric formation along the vessel. Saccular aneurysms occur in the descending thoracic aorta and other locations. Such aneurysms are considered more dangerous since they tend to rupture well below the critical diameters of fusiform recommended for surgery [9]. On the other hand, an intracranial fusiform aneurysm is often associated with ischaemia, mass effect, or bleeding [10,11]. Rupture of fusiform aneurysms, especially those located in the posterior circulation, is often lethal, despite aggressive treatment. Endovascular treatment has been the primary method for vertebral artery fusiform aneurysms (VAFA); however, the risk of treatment cannot be ignored, especially when vital branches are involved [12]. As such, the evaluation of lesion rupture risk and personalized treatment plans for fusiform aneurysms represents an important clinical goal.

Haemodynamic and morphological studies of saccular aneurysms have demonstrated the value of haemodynamic and morphological evaluation in predicting the rupture of the aneurysms [13–15]. However, less work has been done on fusiform aneurysms, possibly due to their more complex morphology. It has been recognized that the morphological characteristics of fusiform aneurysms may play an important role in evaluating rupture tendency [16]. However, manual measurement and assessment on the basis of three-dimensional (3D) reconstruction are time-consuming and prone to error.

Recent advances in machine learning techniques have shown promise that these types of approaches can be highly effective in medical research [17]. Currently, the application of machine learning in the morphological study of cerebrovascular disease is limited. This study represents one of the first attempts to explore the utility of machine learning techniques to evaluate VAFA morphology for the prediction of rupture risk, as well as to assist in decision-making for clinical management of this disease.

## 2. Methods

### 2.1. Patients and image data

A total of 37 patients (23 males, 14 females, mean age = 52.43 years, s.d. = 10.12) with 12 ruptured and 25 un-ruptured aneurysms were included in the study.

All 3D vertebral artery aneurysm images are stored in stereolithography (STL) format files. Each file consists of a triangulated surface of the three-dimensional blood vessel, from which vertex information of the triangles was obtained.

### 2.2. Geometric characteristics

Many observational studies have been conducted to assess the predictors of aneurysm rupture and to guide physicians in decision-making [18–21]. Generally, predictors are categorized into geometric, haemodynamic and clinical characteristics. In this paper, we focus on the relationship between the rupture risk of an aneurysm and geometric properties of an aneurysm and its corresponding parent artery.

The open source software MeshLab [22] was used for processing and editing initial 3D triangular meshes, including segmentation, noise removal and the surface smoothing process. After the initial segmentation process, every 3D image was simplified to a region containing the aneurysm and their parent artery. The refined meshes with removing noise and smoothing surface were exported from MeshLab to be used for further analysis via Matlab [23].

To calculate the geometric properties of aneurysms, the 3D images were first voxelized. Based on the meshes forming the subject's surface, a 3D binary matrix was generated, where the voxels had 3D location information.

The centreline representation of the vessel is simpler than the volume or surface rendering while preserving the topology of the whole vessel trees [24,25]. Thus, we extracted the centrelines of blood vessels and calculated geometric indices based on the centrelines. The 3D medial surface thinning algorithm was used to find the skeleton of the 3D blood vessels [26,27]. In order to obtain a complete centreline, we queued the discrete points that formed the skeleton according to their locations, and then connected the data points by the 3D curve fitting algorithm.

By the definitions of the tangent unit vector, the normal unit vector and the binormal unit vector, together with the Frenet–Serret formulae, we calculated the curvature and the torsion of the centreline. In addition, the cross-sectional profiles and the eight corresponding geometric variables were obtained based on the central path. The geometric indices include maximum, minimum and equivalent diameters (the diameter of a circle with the same area as the region), cross-sectional area, area change rate, eccentricity, solidity (the proportion of the pixels in the convex hull) and extent (the ratio of pixels in the region to pixels in the total bounding box). A detailed explanation of these geometric indices is given in appendix A.

**Figure 1.**
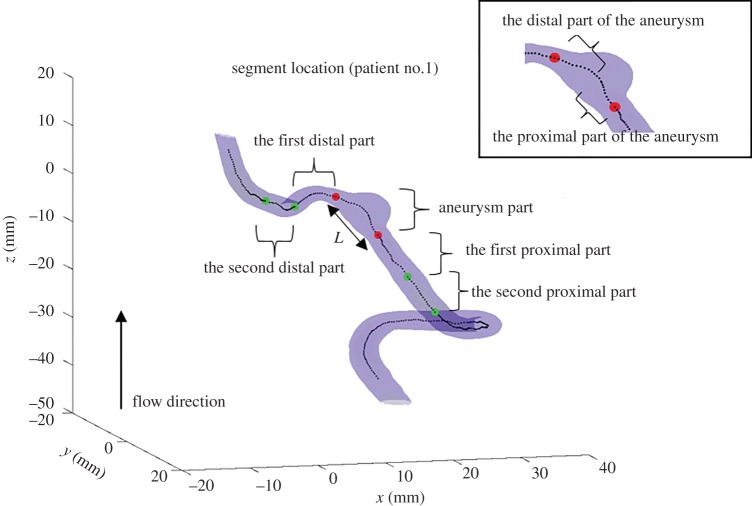
Segment location. Five segments, including the aneurysm part, the first proximal part, the second proximal part, the first distal part, and the second distal part, have the same length *L* (mm). The aneurysm part also consists of the proximal and distal segments, which is shown in the top right corner.

The geometric indices are ten sequences with respect to the centreline location. To analyse these geometric variables more conveniently, we calculated statistical features based on them and used these computed features for the statistical analysis and machine learning classification. Figure 1 shows an aneurysm and its parent artery of patient no. 1. We hypothesized that the aneurysmal shape and its nearby region affected the rupture risk significantly. Hence, we chose five segments with the equal length *L* (mm) as the feature extraction region shown in figure 1, where *L* is the length of an aneurysm, determined based on the cross-sectional area. Furthermore, in order to analyse the aneurysmal shape, the aneurysm part was further divided into two segments for the feature calculation. At these seven segments, the maximal value, mean, standard deviation, integral and variation of the ten geometric value sequences were calculated respectively. We also took the ratios between the values of two segments of the aneurysm part, and the ratios between the aneurysm part, the first distal part and the first proximal part.

Besides the previous features, some features related to the aneurysmal size and shape were considered to be important as well, such as the maximal diameter and the length of the aneurysm, width–length ratio, the angle and the distance between two centreline tangent vectors at the necks of the bulge (refer to figure 6 in the appendix A), and the asymmetry factor. A complete feature list is presented in appendix A.

We computed 571 geometric features in total. The feature set is so large compared to the dataset that over-fitting problem will happen when classifying the aneurysm rupture risk. Therefore, the next step is to analyse and select features by statistical analysis.

### 2.3. Machine learning and statistical analysis

Statistical analysis and machine learning prediction were performed using Matlab. We applied *t*-tests for the geometric features, with a null hypothesis of no interaction between features when separating the classified groups. The *t*-statistics were compared for each feature as a measure of how effective it was at separating groups. Based on the *t*-test, 20 features with the largest *t*-statistics were selected. Feature selection based on their individual ranking may also contain redundant information, so not all features are required. We then checked the correlation of these features and removed 15 redundant features if their Pearson correlation coefficients were above a threshold of 0.5.

After the feature selection process, four typical machine learning algorithms: random forest (RF) [28], support vector machine (SVM) [29], *k*-nearest neighbours (KNN) [30] and subspace discriminant (SD) [31], were applied for classifying the ruptured and un-ruptured cases. A brief description of the machine learning algorithms is presented in appendix B.

Given our relatively small sample size, we used a procedure commonly referred to as a leave-one-out cross-validation to maximize the use of our dataset. This means that of our total sample of 37 patients, we set aside one case to use as the testing set and the remaining 36 cases to use as the training set. The procedure was repeated 37 times, each time using a different case as the test set. This procedure was used for assessing the performance of the four models and therefore choosing the best prediction model.

To validate the classification model, another resampling method train-test split was used for evaluating the performance of the chosen optimal model. Specifically, the whole dataset was randomly divided into a training set (80%) and a test set (20%). The chosen model was trained on the training set and applied to predict the data on the test set. To ensure our model's performance was not biased by a particular data partition, we randomly reassigned the cases into new training and test datasets and repeated the machine learning process 30 times.

## 3. Results

### 3.1. Geometric feature analysis

The highest ranked indices from the feature selection procedure are as follows: the ratio of cross-sectional areas between the proximal and distal parts of the aneurysm (*t*_37_ = 2.6469, *p* = 0.0129), the total maximum diameter at the nearby distal part (*t*_37_ = 2.1634, *p* = 0.0384), the total solidity of the aneurysm (*t*_37_ = 2.0398, *p* = 0.0497), the maximum centreline curvature at the nearby distal part (*t*_37_ = 2.0191, *p* = 0.0519), and the ratio of centreline curvature between the aneurysm and the nearby proximal parts (*t*_37_ = 2.0107, *p* = 0.0531). We set a *p*-value < 0.05 as the criterion for statistical significance.

**Figure 2.**
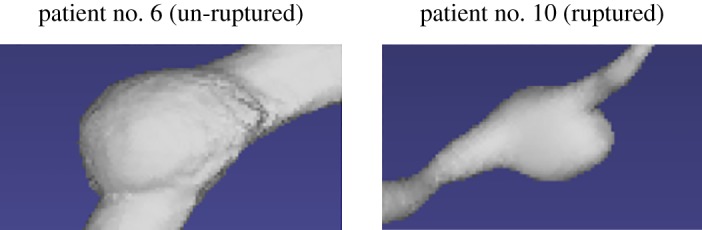
Comparison of aneurysmal shapes. Patient no. 6 is an un-ruptured case shown in the first picture, and the second picture includes a ruptured case. The proximal blood vessels are at the bottom of the pictures.

**Figure 3.**
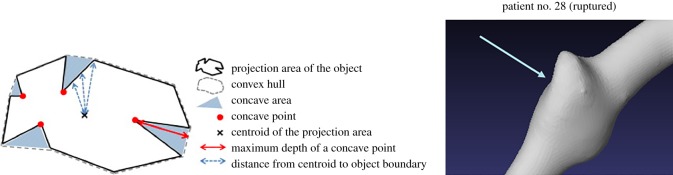
Solidity of an aneurysm. The blue area is the concave area on the left picture [32]. Patient no. 28 is a ruptured case shown on the right, which has a relatively large concave area. The arrow points out the concave region of the aneurysm.

Table 1 shows the relationship between the above five geometric characteristics and rupture in VAFA. The ratio of cross-sectional areas between the proximal and distal parts of an aneurysm represents the shape of an aneurysm. From this table, we find that most of the ruptured aneurysms have smaller ratio values than the un-ruptured cases, meaning that if the proximal part of an aneurysm is smaller than its distal part, the aneurysm has a higher rupture risk. Two cases are taken as the examples to show the comparison of aneurysmal shapes in figure 2. The second geometric index shows that for most ruptured cases, the integral of the maximum diameter of the distal blood vessel near an aneurysm are usually smaller compared to un-ruptured cases. The solidity of an aneurysm is related to the regularity of vascular wall. When an aneurysm has a larger concave area (a smaller solidity value), as shown in figure 3, it is more likely to rupture. The last two features reflect the degree of curvature of the blood vessel near an aneurysm.

**Table 1.** The Relationship between geometric characteristics and rupture in VAFA (*x̄* ± s.d.).

geometric characteristic	rupture	un-rupture	*t*-stat	*p*-value
ratio of cross-sectional areas between the proximal and distal parts of the aneurysm (unitless)	0.95 ± 0.25	1.21 ± 0.34	2.6469	0.0129
total maximum diameter at the nearby distal part (mm^2^)	27.55 ± 14.82	40.76 ± 21.78	2.1634	0.0384
total solidity of the aneurysm (mm)	10.35 ± 2.90	12.88 ± 4.58	2.0398	0.0497
maximum centreline curvature at the nearby distal part (mm^−1^)	0.29 ± 0.12	0.46 ± 0.38	2.0191	0.0519
ratio of centreline curvature between the aneurysm and the nearby proximal part (unitless)	−0.65 ± 0.91	0.72 ± 3.16	2.0107	0.0531

### 3.2. Classification

**Figure 4.**
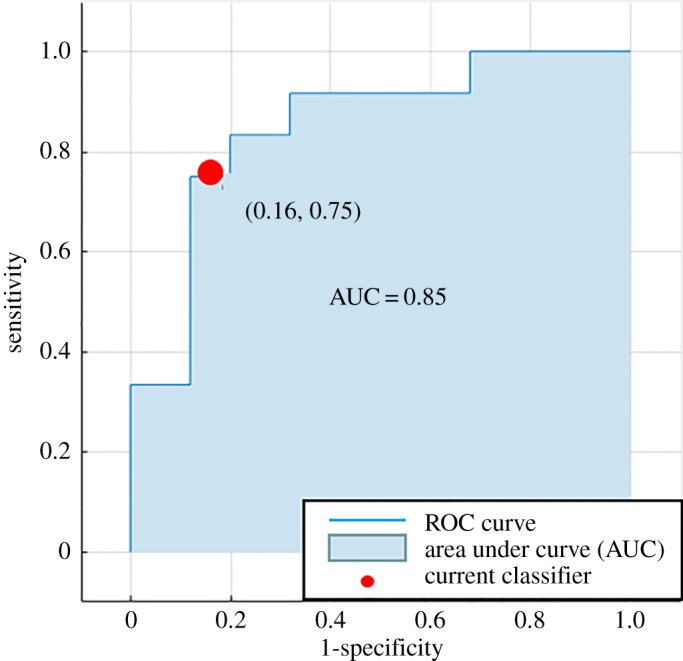
ROC curve produced by SVM. The blue area indicates the area under the curve (AUC). The optimal operating point on the ROC curve is denoted by the red point (0.16, 0.75).

**Figure 6.**
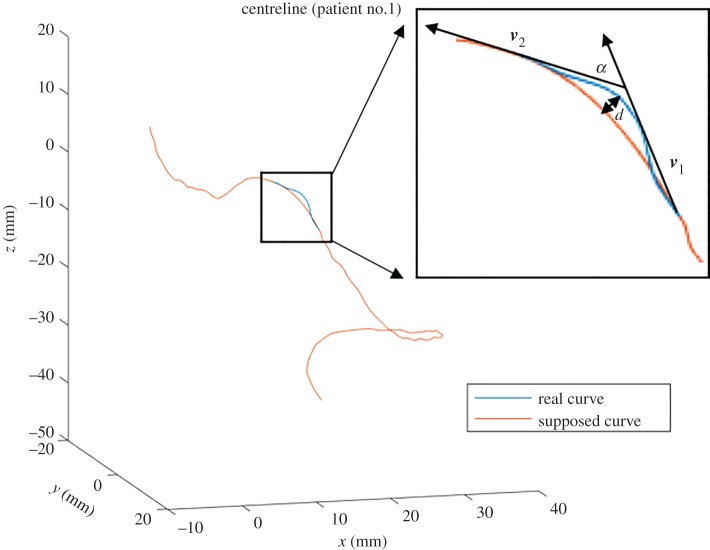
Centreline. Real curve is the centreline of an aneurysm, and the supposed curve is the centreline of the normal blood vessel. The aneurysm part is zoomed in and shown in the small black box. In the black box, the two rays are centreline tangent vectors at the necks of the bulge, and the maximum distance of two curves is denoted by *d*.

Based on the leave-one-out cross-validation method, the comparison results of four machine learning models are shown in table 2. The model with the lowest overall error estimate is SVM, with an average accuracy of 81.08%. To compare the models, we performed a hypothesis test on the performance difference of each model, using the SVM as the baseline. The results show that although SVM performed better than other models, the difference between model classification performance was not statistically significant. This is most likely due to the relatively small size of the data. Figure 4 depicts the receiver operating characteristics (ROC) curve produced by SVM. The area under the curve (AUC) is 0.85. The optimal operating point on the ROC curve shows the sensitivity of the classifier SVM is 75%, and the specificity achieves 84%.

**Table 2.** Comparison of four models (leave-one-out).

machine learning algorithm	accuracy	*t*-statistic	*p*-value
SVM	81.08%		
RF	72.92%	0.8216	0.4141
KNN	75.68%	0.5583	0.5784
SD	72.97%	0.8216	0.4141

We further evaluated the performance of the methods using the train-test data split. From table 3, we find that the performance difference between these methods was not statistically significant, but the SVM model still performed better than others. It achieved an average classification accuracy of 81.43% (±13.08%) on the test set.

**Table 3.** Comparison of four models (train-test data split).

machine learning algorithm	accuracy	*t*-statistic	*p*-value
SVM	81.43 (±13.08) %		
RF	76.67 (±13.78) %	1.3732	0.1750
KNN	79.05 (±14.88) %	0.6583	0.5130
SD	79.52 (±13.88) %	0.5471	0.5864

## 4. Discussion

The objectives of our study were to use clinical data of the patients to evaluate a set of geometric characteristics describing the size and shape of an aneurysm and nearby blood vessel information and to apply a collection of machine learning models to discriminate ruptured and un-ruptured aneurysms. The rapid pace at which medical image data are being generated has resulted in a gap between the collection of data and analysis for decision-making. Machine learning combined with image processing techniques can help in identifying potentially useful patterns in image data and build models that are capable of predicting rupture risk of an aneurysm based on the known patient data.

The results of this study show that some geometric indices related to the size and shape of an aneurysm and nearby blood vessel were correlated with rupture risk. The most effective features for our current data include the cross-sectional area change of an aneurysm, the total maximum diameter of the nearby distal blood vessel, the total solidity of an aneurysm, the maximum curvature of the nearby distal blood vessel, and the ratio of curvature between an aneurysm and the nearby proximal blood vessel. These geometric variables are suitable for further study with more image data. However, some geometric indices such as length, width and width–length ratio of the aneurysm, which are effective for distinguishing saccular aneurysms, we deemed to be not statistically significant for predicting the rupture risk of VAFA (*t*_37_ = 1.7301 (*p* = 0.0943), *t*_37_ = 0.6647 (*p* = 0.5123) and *t*_37_ = 0.9036 (*p* = 0.3742), respectively).

Due to the complexity of fusiform aneurysm morphology, manual measurement of geometric indices from 3D images is a difficult and time-consuming task. In our study, we extracted geometric information automatically by image processing techniques, which have the benefit of being much faster and with improved accuracy as compared to manual measurements. For the current dataset, the overall classification accuracy achieved 81%, much higher than chance (50%). The performance including sensitivity and specificity of the SVM classifier also proved that machine learning is an efficient method to differentiate ruptured and un-ruptured cases.

Although machine learning is transforming modern medicine and was proved to be useful for our study, we still need to consider its limitations. First, machine learning learns through historical data. The bigger the data and the longer it is exposed to these data, the better it will perform. Our current dataset is relatively small, so the geometric characteristics and the machine learning model need to be further validated on a larger dataset. Second, machine learning systems cannot always provide rational reasons for a particular prediction or decision. Thus, human collaboration is necessary to better evaluate the outputs of these systems.

## 5. Conclusion

In this paper, we have brought image analysis, statistical analysis and machine learning techniques together to study VAFA morphology, thereby predicting the rupture risk of VAFA. This method extracted multiple geometric characteristics automatically and accurately from the complex morphology of VAFA. Hypothesis testing and machine learning models further validated that some of these geometric indices were effective and meaningful predictors for the rupture risk of VAFA. Our results suggest the popular SVM machine learning classification technique can be used to classify ruptured and un-ruptured aneurysms successfully with high accuracy. Therefore, we conclude that studying the relationship between geometric characteristics and rupture in VAFA and thereby predicting the rupture risk via machine learning techniques is a promising research direction.

The application of machine learning algorithms to 3D medical image analysis is still in its infancy. However, machine learning combined with geometric feature extraction as a non-invasive method has great potential as an early predictor of the rupture risk, and ultimately provides a useful tool for the personalized treatment of VAFA. In addition, our approach can be extended further for studying 3D medical images of the other type of aneurysms and serve as a useful risk predictor for researchers and surgeons.

## Figures and Tables

**Figure 5.**
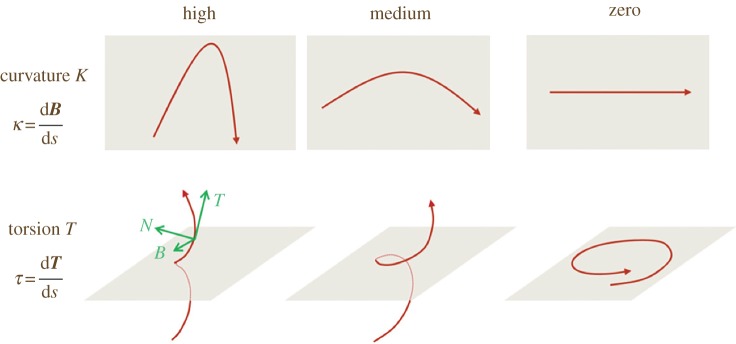
Curvature and torsion. The first row shows different curvature, and the second row shows different torsion [33].

## Data accessibility

Data supporting this paper are openly available at the GitHub page: https://github.com/ngold5/aneurysm_classification.

## Appendix A. Definition of geometric features

By the definitions (A.1–A.3) of the tangent unit vector ***T***, the normal unit vector ***N*** and the binormal unit vector ***B***, together with the Frenet–Serret formulae (A.4), we have the curvature *κ* and the torsion *τ* of the centreline. To reduce the influence of the unsmooth centreline, the sliding window strategy was also involved to calculate the curvature and the torsion.

A.1 $$T=\frac{dr/ds}{∥dr/ds∥},$$

where ***r*** is a curve in Euclidean space, and *s* represents the arc length.

A.2 $$N=\frac{dT/ds}{∥dT/ds∥},$$

A.3 B=T×N

and

A.4 $$\left[\begin{array}{c} T^{\prime }\\ N^{\prime }\\ B^{\prime } \end{array}\right]=\left[\begin{array}{ccc} 0 & \kappa & 0\\ -\kappa & 0 & \tau \\ 0 & -\tau & 0 \end{array}\right]\;\left[\begin{array}{c} T\\ N\\ B \end{array}\right].$$

Based on the curvature and torsion of the centreline, we found the central path and therefore calculated another eight geometric variables. The concrete explanation of these 10 geometric indices are shown as follows.

**A.1. Centreline curvature *κ* (mm^−1^)**

Curvature *κ* represents the rate of change of the angle through which the tangent to a curve turns in moving along the curve. The curvature *κ* is the solution of the Frenet–Serret formulae (A.4).

**A.2. Centreline torsion *τ* (mm^−1^)**

In three dimensions the torsion *τ* of a curve measures how sharply it is twisting out of the plane of curvature. The torsion *τ* is solved by the Frenet–Serret formulae (A.4). Figure 5 shows some examples of curvature and torsion.

**A.3. Cross-sectional area *A* (mm^2^)**

The cross-sectional area *A* is the area of a two-dimensional shape that is obtained when a three-dimensional object. Along the centreline, a series of slices containing the cross-sectional profiles are obtained. The cross-sectional area *A* is calculated by the number of pixels in the region.

**A.4. Rate of change of cross-sectional area ACR (mm)**

The rate of change of cross-sectional area ACR is the change of the cross-sectional area with respect to the position change at the centreline *r*.

$$\mathrm{ACR}=\frac{dA}{dr}.$$

**A.5. Maximal diameter Max*D* (mm)**

The maximal diameter Max*D* is the maximal distance between the centreline and the blood vessel surface.

**A.6. Minimal diameter Min*D* (mm)**

The minimal diameter Min*D* is the minimal distance between the centreline and the blood vessel surface.

**A.7. Equivalent diameter Equiv*D* (mm)**

Equivalent diameter Equiv*D* is the diameter of a circle with the same area as the region.

$$\mathrm{Equiv}D=\sqrt{\frac{4A}{\pi }}.$$

**A.8. Eccentricity *e* (unitless)**

The eccentricity *e* is the ratio of the distance between the foci *f* of the ellipse and its major axis length Max*D*. The value is between 0 and 1. An ellipse whose eccentricity is 0 is a circle, while an ellipse whose eccentricity is 1 is a line segment.

$$e=\frac{\;f}{\mathrm{Max}D}.$$

**A.9. Solidity SLD (unitless)**

The solidity SLD is the proportion of the pixels in the convex hull.

$$\mathrm{SLD}=\frac{A}{A_{\mathrm{convex}}},$$

where *A*_convex_ is the area of the smallest convex polygon that can contain the region.

**A.10. Extent EXT (unitless)**

The extent EXT is the ratio of pixels in the region to pixels in the total bounding box.

$$\mathrm{EXT}=\frac{A}{A_{\mathrm{bb}}},$$

where *A*_bb_ is the area of the bounding box that can contain the region.

In addition to the above features, some geometric indices are important as well. The maximal diameter *D*_max_ (mm), the length *L* (mm), the proximal neck diameter *D*_neck,p_ (mm), the distal neck diameter *D*_neck,d_ (mm) of the aneurysm, and the ratios between these four factors and the normal diameter of the parent vessel *D*_normal_ (mm) were used to represent the aneurysmal size.

To describe the aneurysmal shape, we introduced an imaginary curve to represent the centreline of the normal vessel at the aneurysm region and compared it with the centreline of an aneurysm since there was no information on the changes of morphometric characteristics regarding an aneurysm. The hobby curve, a spline interpolation algorithm, was applied to connect the centrelines at the necks of the bulge [34].

Figure 6 depicts the real centreline of an aneurysm and the supposed centreline of the normal blood vessel. The distance between the real and supposed curves was calculated, and the maximum value, the accumulated value, the average, the standard deviation and the variation of the distance were taken as the geometric features. The angle and the distance between two centreline tangent vectors at the necks of the bulge, shown in figure 6, and the asymmetry factor $\left(1-(d/D_{\max})\right)$ are also the features for the further analysis.

## Appendix B. Brief description of machine learning algorithms

**B.1. Random forest**

Random forest (RF) [28] is an ensemble learning method for classification, that operates by constructing a multitude of decision trees at training time and outputting the class that is the mode of the classes of the individual trees. Random forest corrects for decision trees' habit of over-fitting to their training set.

**B.2. Support vector machine**

Support vector machine (SVM) [29] classifies data by finding the best hyperplane that separates data points of one class from those of the other class. The best hyperplane for an SVM is defined as the hyperplane with the largest margin between the two classes; that is the maximal width of the slab parallel to the hyperplane that has no interior data points.

**B.3. *k*-Nearest neighbours**

The *k*-nearest neighbours algorithm (KNN) [30] is a non-parametric method used for classification. Given a set *X* of *n* points and a distance function, *k*-nearest neighbours search finds the *k* closest points in *X* to a query point or set of points. Various metrics can be used to determine the distance. KNN-based algorithms are widely used as benchmark machine learning rules.

**B.4. Subspace discriminant**

Subspace discriminant (SD) [31] is a high-quality ensemble model composed of a combination of multiple linear discriminant classifiers, where a random subset of the predictors is for each learner. The linear discriminant analysis assumes that different classes generate data based on different Gaussian distributions [31]. To train a classifier, the fitting function estimates the parameters of a Gaussian distribution for each class.
